# Identification of truncated variants in *GLI family zinc finger 3* (*GLI3*) associated with polydactyly

**DOI:** 10.1186/s13018-024-04928-0

**Published:** 2024-07-30

**Authors:** Run-Yan Wang, Qin Xiong, Si-Hua Chang, Jie-Yuan Jin, Rong Xiang, Lei Zeng, Fang Yu

**Affiliations:** 1 Department of Hand and Microsurgery, Xiangya Hospital, Central South University, Changsha, 410000 China; 2 School of Life Sciences, Central South University, Changsha, 410000 China; 3 National Clinical Research Center for Geriatric Disorders, Xiangya Hospital, Central South University, Changsha, 410000 China; 4 Microsurgery & Reconstruction Research Center, Xiangya Hospital, Central South University, Changsha, 410000 China

**Keywords:** Polydactyly, GLI3, Truncated variant, Isolated polydactyly, Greig cephalopolysyndactyly syndrome

## Abstract

**Background:**

Polydactyly is a prevalent congenital anomaly with an incidence of 2.14 per 1000 live births in China. *GLI family zinc finger 3* (*GLI3*) is a classical causative gene of polydactyly, and serves as a pivotal transcription factor in the hedgehog signaling pathway, regulating the development of the anterior-posterior axis in limbs.

**Methods:**

Three pedigrees of polydactyly patients were enrolled from Hunan Province, China. Pathogenic variants were identified by whole-exome sequencing (WES) and Sanger sequencing.

**Results:**

Three variants in *GLI3* were identified in three unrelated families, including a novel deletion variant (c.1372del, p.Thr458GlnfsTer44), a novel insertion-deletion (indel) variant (c.1967_1968delinsAA, p.Ser656Ter), and a nonsense variant (c.2374 C > T, p.Arg792Ter). These variants were present exclusively in patients but not in healthy individuals.

**Conclusions:**

We identified three pathogenic *GLI3* variants in polydactyly patients, broadening the genetic spectrum of *GLI3* and contributing significantly to genetic counseling and diagnosis for polydactyly.

**Supplementary Information:**

The online version contains supplementary material available at 10.1186/s13018-024-04928-0.

## Background

Polydactyly is a common congenital limb anomaly, occurring in approximately 2.14 per 1000 live births in China [1]. Polydactyly includes syndromic polydactyly and isolated polydactyly (IPD). IPD is further classified into three categories: preaxial polydactyly, which involves a non-functional duplicated thumb on the hand or foot; postaxial polydactyly, featuring a fully or partially duplicated digit on the ulnar side of the hand or foot; and central polydactyly, a less common type involving anomalies of the index, middle, or ring fingers [2]. Surgical interventions, such as removal of supernumerary digits and redundant skeletal structures, are the primary treatments to maintain joint stability and soft tissue balance [3]. Various genes have been identified as contributing to IPD, including the *GLI family zinc finger 3* (*GLI3*, OMIM 165,240), *zinc finger protein 141* (*ZNF141*, OMIM 194,648), *IQ domain-containing protein E* (*IQCE*, OMIM 617,631), *GLI family zinc finger 1* (*GLI1*, OMIM 165,220), *Family with sequence similarity 92*,* member A* (*FAM92A*, OMIM 617,273), *KIAA0825* (OMIM 617,266), *Dachshund family transcription factor 1* (*DACH1*, OMIM 603,803), *Mirror-image polydactyly 1* (*MIPOL1*, OMIM 606,850), and *Paired-like homeodomain 1* (*PITX1*, OMIM 602,149) [4–12]. Notably, *GLI1* and *GLI3* are crucial for limb development, with *GLI3* being a classical gene implicated in polydactyly [13].

The *GLI3* gene, located on chromosome 7p14.1, encodes an 1580-amino acid protein that exists in two isoforms: the full-length activator (GLI3-A) and the truncated repressor (GLI3-R) [14]. The GLI3 protein plays a pivotal role in limb development by acting as a nuclear transducer and negative regulator of Soni Hedgehog (SHH) signaling, which establishes the zone of polarizing activity essential for the formation of the anterior-posterior limb axis [15, 16]. Loss-of-function variants in *GLI3* can lead to various limb development disorders, including Greig cephalopolysyndactyly syndrome (GCPS; OMIM 175,700), preaxial polydactyly type A/B (PAPA/PAPB; OMIM 174,200), Pallister Hall syndrome (PHS; OMIM 146,510), postaxial polydactyly type A1 and B (OMIM 174,200) and preaxial polydactyly type III (OMIM 174,700) [17–20].

In this study, we recruited several families with polydactyly and identified three *GLI3* variants, including a novel deletion variant (NM_000168.6: c.1372del, p.Thr458GlnfsTer44), a novel insertion-deletion (indel) variant (NM_000168.6: c.1967_1968delinsAA, p.Ser656Ter), and a previously reported nonsense variant (NM_000168.6: c.2374 C > T, p.Arg792Ter). These findings broaden the genetic spectrum associated with *GLI3* and enhance genetic counseling and diagnosis of polydactyly.

## Methods

### Ethical compliance

This study was approved (2,023,030,444) by the Ethics Committee of Xiangya Hospital, Central South University, Changsha, China. We performed this study in accordance with the principles outlined in the Declaration of Helsinki. The patients/participants or their guardians provided written informed consent to participate in the study.

### Participants/patients

Three families (Family I-III) were investigated in this study. Peripheral blood samples were collected from probands and their family members. Clinical data were recorded carefully.

### Whole-exome sequencing

Genomic DNA was extracted using a DNeasy Blood and Tissue Kit (Qiagen, Valencia, CA, USA). Exome capture and whole-exome sequencing (WES) were conducted at Berry Genomics (Beijing, China). 1 µg DNA was captured using the SureSelect Human All Exon Kit V6 (Agilent Technologies, Inc., CA, USA) and sequenced using the Illumina HiSeq4000 platform (Illumina Inc., CA, USA). Briefly, the genomic DNA was randomly extracted using a Covaris S220 sonicator (Covaris, Inc., MA, USA). The fragmented DNA underwent three enzymatic steps: end repair, a-tailing, and adapter ligation. The adapter-ligated DNA fragments were amplified using Herculase II Fusion DNA Polymerase (Agilent). Finally, the exosomes in the pre-capture libraries were captured using the SureSelect capture library kit (Agilent). After DNA quality assessment, the captured DNA library was subjected to WES on the Illumina HiSeq4000 platform. Downstream processing was carried out using the Genome Analysis Toolkit (GATK), Varscan2, and Picard, and variant calls were made with the GATK Haplotype Caller 12. Variant annotation was performed according to Ensemble release 82, and filtering was conducted using ANNOVAR Documentation.

The filtering strategies conformed to those used in our previous study [21]. Variants with an alternative allele frequency > 0.001 in the 1000G database (http://www.1000genomes.org/) and the GnomAD database (https://gnomad.broadinstitute.org/) were used for further analysis. These filtered variants were predicted their pathogenicity using MutationTaster (http://www.mutationtaster.org/), Polyphen-2 (http://genetics.bwh.harvard.edu/pph2/), SIFT (http://provean.jcvi.org/index.php), and CADD (https://cadd.gs.washington.edu/snv) [22]. Bone development related genes were used to filter candidate variants [23].

### Co-segregation analysis

Co-segregation analysis was performed on each family member using Sanger sequencing. The primer pairs (GLI3-1-F: 5’-CCTCCTGTTGTGTCTGATTCTT-3’; GLI3-1-R: 5’-GGTTCCTGAATACCATCCACTT-3’; GLI3-2-F: 5’-GAGGCTCATGTCACCAAGAA-3’; GLI3-2-R: 5’-CTGTGAAGTCAGAAGGAGAGTG-3’; GLI3-3-F: 5’-CCAAATGGATGGAGCACGTA-3’; GLI3-3-R: 5’-CGGATGGTTACAGCGTCATT-3’) used for PCR amplification were designed using Primer 5. The sequences of the PCR products were determined using an ABI 3100 Genetic Analyzer (ABI, Foster City, CA, USA) [22].

## Results

### Clinical description

**Fig. 1:**
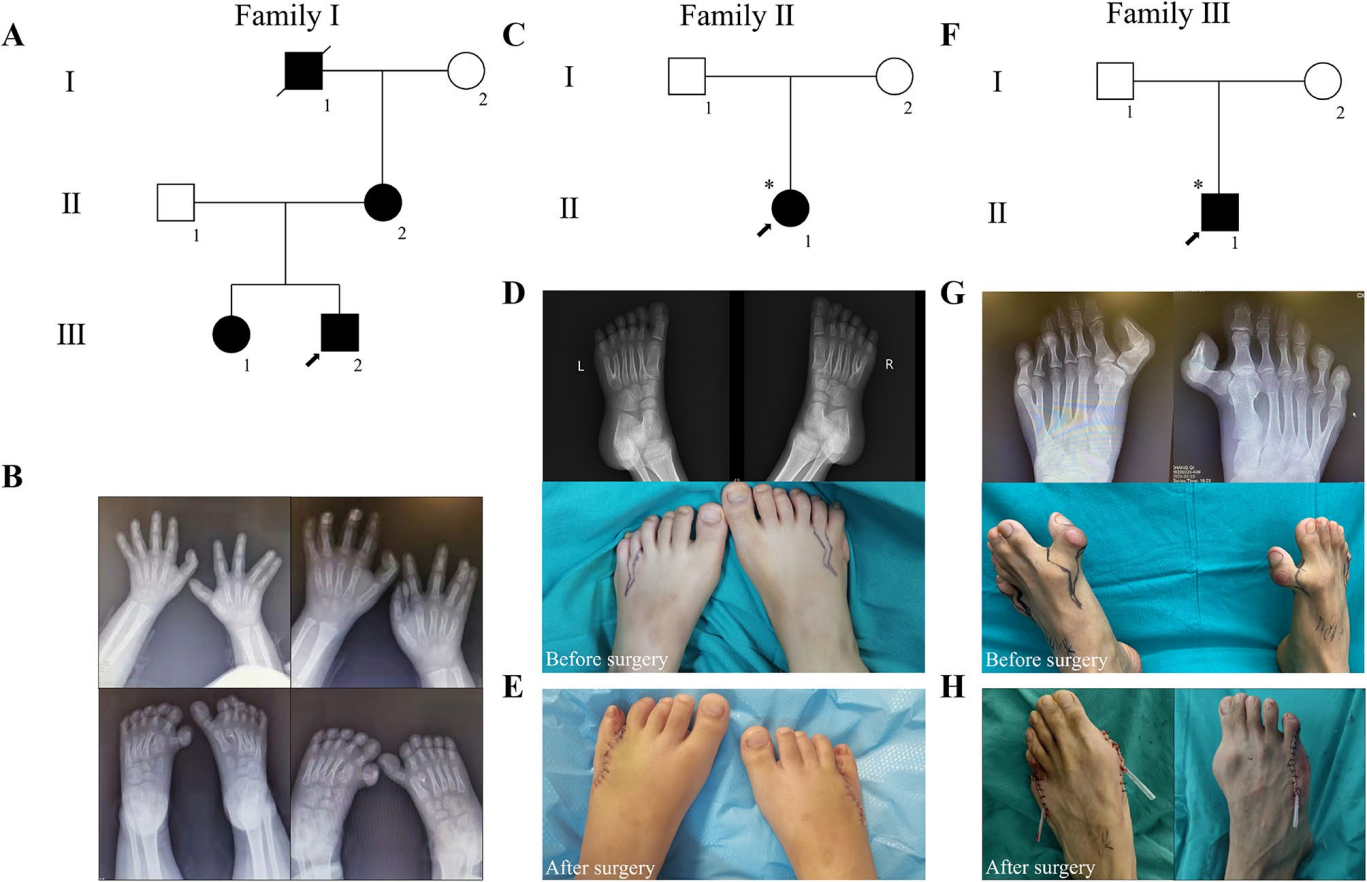
Clinic description of the probands with polydactyly related to GLI3. (**A**) Pedigree of Family I. (**B)** Clinical features of proband I. (**C**) Pedigree of Family II. (**D**) Clinical features of proband II before surgery. (**E**) Clinical features of proband II after surgery. (**F**) Pedigree of Family III. (**G**) Clinical features of proband III before surgery. (**H**) Clinical features of proband III after surgery. Squares = men; circles = women; black symbols = individuals with variants; Slashes = individuals who die; arrows = the probands

We conducted an investigation into three Chinese families (Family I-III) from Hunan Province of China (Table 1). Family I consists of six immediate members, four of whom have exhibited features of polydactyly or syndactyly (Fig. 1A). Radiography showed that the proband presented syndactyly of the fourth and fifth fingers on the right hand and polydactyly of thumbs on each foot (Fig. 1B). Additionally, his sister was affected by syndactyly on all limbs, and his mother had surgically corrected dysmorphic toes. The family reported a history of polydactyly in the deceased grandfather’s toes.

**Table 1:** Clinical details of patients in this study

		Family I				Family II	Family II
		Proband I	Mother	Grandfather	Sister	Proband II	Proband III
Upper limb	Polydactyly	-	-	-	-	Bil	-
	Syndactyly	Bil (4-5)	-	-	Bil	-	-
Lower limb	Polydactyly	Bil (1)	Bil	Bil	-	Bil	Bil (1,5)
	Syndactyly	-	-	-	Bil	-	Bil (1-2)

Bil, bilateral; 1, thumb or toe; 2/4/5, the second/fourth/fifth finger

Proband II from Family II was born with polydactyly affecting both hands and feet, with no similar conditions reported in the family’s history (Fig. 1C and D). Surgical removal of the extra fingers was performed at a different hospital when the patient was one year old, and subsequent toe resection occurred at our hospital, resulting in successful recover (Fig. 1E).

Proband III from Family III displayed heptadactyly on both feet, polydactyly on the first and fifth toes, and syndactyly between the first and second toes, with no reported familial history of these anomalies (Fig. 1F and G). The extra toes were surgically removed (Fig. 1H). Considering the clinical manifestations in Family I and III, we made primary diagnosis of mild GCPS, whereas the proband from Family II appeared to have IPD [13].

### Genetic analysis

Using WES and Sanger sequencing, we identified three *GLI3* variants in the probands from three families, including two novel variants (NM_000168.6: c.1372del, p.Thr458GlnfsTer44 and c.1967_1968delinsAA, p.Ser656Ter) and one known variant (NM_000168.6: c.2374 C > T, p.Arg792Ter). According to the standards of the American College of Medical Genetics and Genomics (ACMG) standards, these variants were designated as “pathogenic” (Table 2).

**Table 2:** Information and pathogenicity classification of *GLI3* variants in this study

Patient	Gene	Variant	Pathogenicity prediction	GnomAD	1000G	OMIM clinical phenotype	American College of Medical Genetics classification
Proband I	*GLI3*	NM_000168.6: c.1372del, p.Thr458GlnfsTer44	MutationTaster: D Polyphen-2:- SIFT: - CADD: -	-	-	AD; Polydactyly, postaxial, types A1 and B; Polydactyly, preaxial, type IV	Pathogenic (PVS1, PM2, PP1, PP3)
Proband II	*GLI3*	NM_000168.6: c.1967_1968delinsAA, p.Ser656Ter	MutationTaster: D Polyphen-2: - SIFT: - CADD: -	-	-		Pathogenic (PVS1, PM2, PP3)
Proband III	*GLI3*	NM_000168.6: c.2374 C > T, p.Arg792Ter	MutationTaster: D Polyphen-2:- SIFT: - CADD: 37	-	-		Pathogenic (PVS1, PM2, PP3)

D, disease causing; AD, autosomal recessive

**Fig. 2:**
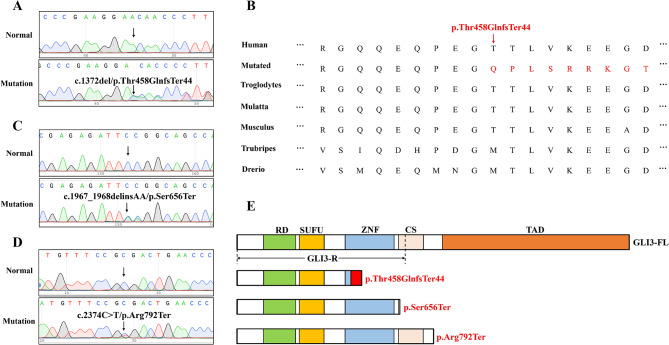
Genetic description of the probands with polydactyly related to GLI3. (**A**, **C**,** D**) Sanger sequencing results of the GLI3 variant among the probands. (**B**) Alignment analysis of the region (p.458–466) in the GLI3 amino acid sequence showed that the region was highly conserved. (**E**) Localization of the variant in GLI3. Red region = frameshift variant; RD = repressor domain; SUFU = SUFU site; ZFN = zinc finger domain; CS = cleavage site, TAD = transactivation domain

In Family I, WES produced 9.7 Gb of data, achieving 98.1% coverage of the target region and over 10× coverage for 99.0% of the targets. After excluding common variants in the 1000G and GnomAD databases, and retaining variants predicted to be disease-causing using MutationTaster, Polyphen-2, SIFT and CADD, which were also positioned in genes associated with bone development, one *GLI3* variant (c.1372del, p.Thr458GlnfsTer44) was identified. Sanger sequencing further confirmed the heterozygous frameshift variant in *GLI3* in Proband I (Fig. 2A). Adherence to ACMG guidelines, this variant was deemed “pathogenic”: it was a frameshift variant often leading to loss of function (PVS1), was absent in control cohorts from the 1000G and GnomAD databases (PM2) and co-segregated with the polydactyly phenotype in Family I (PP1). It was also predicted to have a deleterious effect on the gene product as determined by tools such as MutationTaster (PP3). Additionally, alignments of GLI3 amino acid sequences across various species showed high conservation in this region (p.458–466) (Fig. 2B), demonstrating the critical nature of this sequence.

In Family II, we identified the heterozygous nonsense variant (c.1967_1968delinsAA, p.Ser656Ter) in Proband II (Fig. 2C). According to the ACMG guidelines, this variant was classified as a nonsense variant (PVS1). It has not been observed in healthy population databases (PM2), and bioinformatic tools predict its deleterious effects (PP3). Consequently, it was designated as “pathogenic”.

In Family III, a heterozygous variant (c.2374 C > T, p.Arg792Ter) was identified, which was a known truncation variant (Fig. 2D) [24]. Similarly, according to the ACMG guidelines (PVS1, PM2, PP3), this variant was considered “pathogenic”.

## Discussion

GLI3 is a pivotal transcription factor in the SHH signaling pathway, comprising multiple domains: a repressor domain (RD), a suppressor of fusion (SUFU) site, a zinc finger domain (ZFN), a cleavage site (CS), a CREB-binding protein (CBP) domain, and two transactivation domains (TAD1 and TAD2). The ZFN domain facilitates GLI3’s DNA binding, enabling transcriptional suppression or activation of SHH target genes. Additionally, the CS domain allows GLI3 to be cleaved from GLI3-A to GLI3-R by the proteasome [25–28]. In this study, we identified three *GLI3* variants (c.1372del, p.Thr458GlnfsTer44, c.1967_1968delinsAA, p.Ser656Ter, and c.2374 C > T, p.Arg792Ter) in polydactyl y patients. The variant p.Thr458GlnfsTer44, located in the ZFN domain, disrupts transcriptional functions, while variants p.Ser656Ter and p.Arg792Ter, positioned in or adjoining the CS, directly influence the GLI3-A to GLI3-R conversion (Fig. 2E).

It has been suggested that the location of *GLI3* variants significantly impacts the manifestation of digital anomalies due to the dual role of GLI3 as an activator or repressor in the SHH pathway [29]. In mouse model, GLI3 haploinsufficiency, often resulting from loss-of-function variants upstream of or within the ZFN domain, has been linked to the pathogenesis of GCPS, characterized by cranial enlargement and a wide interorbital distance [30]. In this study, the frameshift variant p.Thr458GlnfsTer44 in Family I introduces a premature stop codon, leading to a GLI3 protein lacking the essential ZFN domain for DNA binding, which may contribute to GCPS, corroborating earlier research findings. The CS domain is critical in regulating the ratio of GLI3-A/GLI3-R, thus, variants in the CS region can result in an excess of GLI3-R in limb buds and the neural tube, which predominantly drives the pathogenesis of PHS. PHS is associated with diverse anomalies including hypothalamic hamartoma, cleft larynx, imperforate anus, and pulmonary lobation anomalies [31]. This study also identified two truncation variants (p.Ser656Ter and p.Arg792Ter) within the CS domain, which may result in premature termination of GLI3, loss of GLI3-A function, and excessive production of GLI3-R (Fig. 2E). Notably, while the patient in Family II was diagnosed with IPD, and the patient in Family III with mild GCPS, neither presented craniofacial anomalies. Research by Sczakiel et al. indicated that GLI3 variants linked to IPD can also occur in the ZFN and CS domains, challenging previous beliefs that such variants are confined to the C-terminal TAD domain, aligning whit our findings in Family II [32].

A previous study proposed that GLI3 expression could demarcate between posterior and anterior hand anomalies. Specifically, GLI3 haploinsufficiency has been linked to the etiology of preaxial polydactyly, whereas postaxial polydactyly arises from abnormal truncations in the TAD [33]. In this study, Proband I, exhibited preaxial polydactyly traits, carried a truncating variant upstream of the ZFN in GLI3, likely leading to GLI3 haploinsufficiency. Conversely, Proband II, who displayed postaxial polydactyly, harbored a truncating variant in the CS domain of GLI3, potentially resulting in TAD functional deficiency. These observations align with findings from Bass et al. However, despite the variant in Proband III being associated with a TAD deficiency, the phenotype included both preaxial and postaxial polydactyly, highlighting gaps in current understanding of the relationship between *GLI3* variant locations and clinical manifestations. Future research is warranted to elucidate this association more comprehensively.

**Fig. 3:**
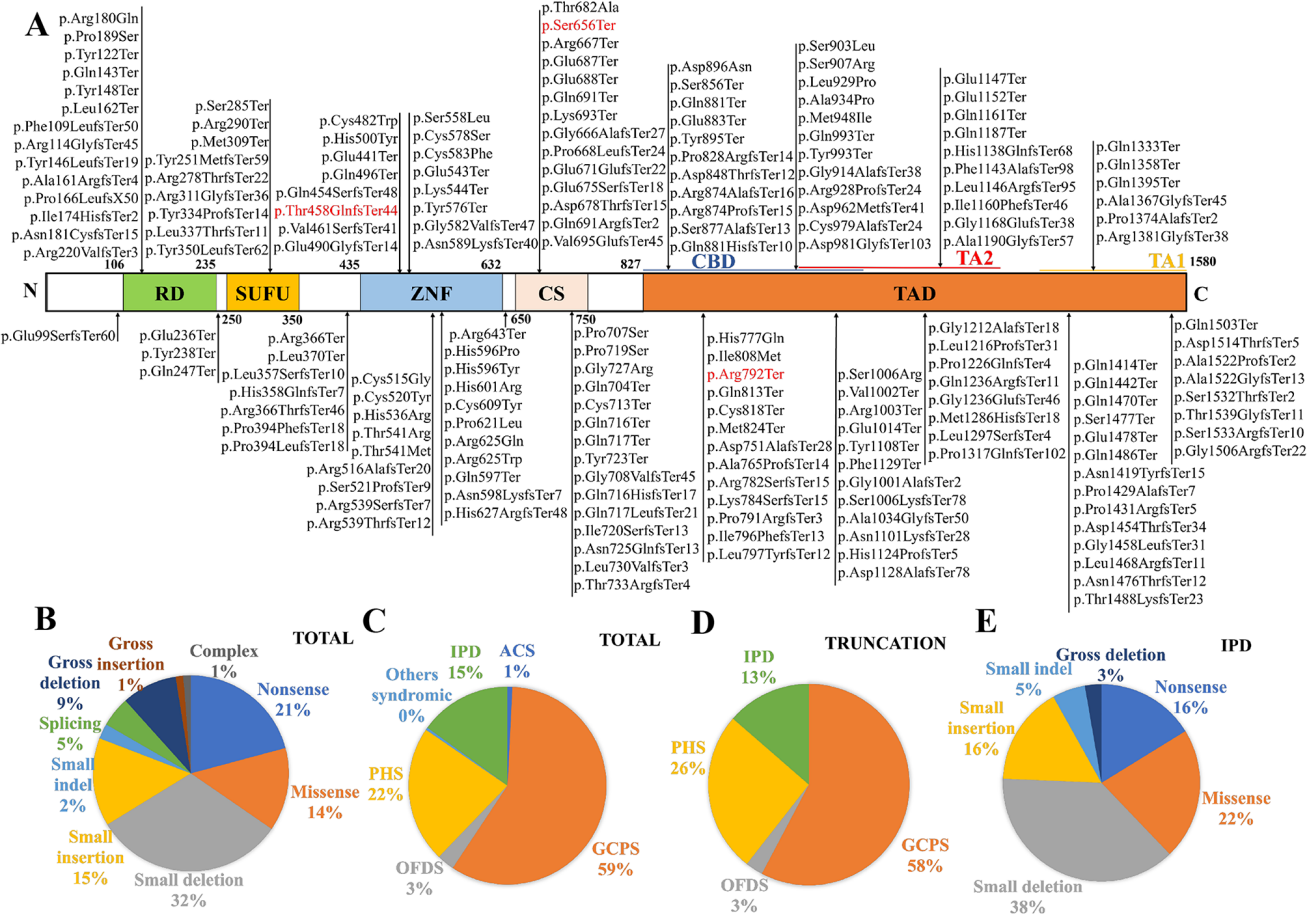
Analysis of polydactyly related to GLI3 reported in other studies. (**A**) Variants of the GLI3 protein have been reported. (**B**) Types of GLI3 variants involved in polydactyly. (**C**) Polydactyly types caused by GLI3 variants. (**D**) Polydactyly types caused by GLI3 truncation variants. (**E**) Types of GLI3 variants involved in isolated polydactyly. RD = repressor domain; SUFU = SUFU site; ZFN = zinc finger domain; CS = cleavage site; CBD = CREB-binding protein (CBP) domain; TAD = transactivation domain; TA1 = transactivation domain 1; TA2 = transactivation domain 2

To investigate the genotype-phenotype correlation further, we analyzed 246 cases of polydactyly linked to *GLI3* variants (Fig. 3A). A predilection for variants in the C-terminal region of GLI3 was noted. Statistical analyses confirmed that the majority of polydactyly cases resulted from by loss-of-function *GLI3* variants (Fig. 3B), with 144 cases (approximately 59%) associated with GCPS (Fig. 3C), corroborating previous studies [13]. In this study, both Family I and III demonstrated features of GCPS. Furthermore, prior research estimated the incidence of IPD at 1.01 per 1000 live births [34], and our analysis revealed that a only 13% of IPD cases involved truncating GLI3 variants (Fig. 3D). In this study, all patients exhibited GLI3 truncations, yet the patients in Family II was diagnosed with IPD, whereas patients in Family I and III showed milder forms of GCPS, primarily affecting hands or feet. These findings suggest a tendency for milder phenotypic manifestations among GLI3 truncation carriers in Hunan Province. Remarkably, a further statistical assessment of all IPD patients confirmed that loss-of-function *GLI3* variants are also a predominant pathogenic factor (Fig. 3E).

In this study, surgical procedures were performed on patients affected by polydactyly to remove the supernumerary fingers. This process may result in damage to the muscles and bones in the affected areas, impacting the patients’ daily lives during the recovery period. Recent studies have identified multiple genes associated with susceptibility to skeletal muscle injury and repair capacity [35–40], and significant differences in single nucleotide polymorphisms (SNPs) of these genes have been observed among populations with different levels of physical activity [41, 42]. These findings suggest that analyzing specific SNPs of susceptibility genes in patients could be beneficial in predicting their postoperative recovery capacity. For patients with high muscle susceptibility and low repair capacity, it may be advisable to consider therapeutic measures that promote muscle repair during surgery. For example, based on the ability of mesenchymal stem cells (MSCs) to differentiate into various mesenchymal tissues, applying MSCs to the injury site may significantly improve the biomechanical properties, structure, and function of the muscle postoperatively [43].

## Conclusions

In conclusion, we identified a novel frameshift variant (c.1372del, p.Thr458GlnfsTer44), a novel indel variant (c.1967_1968delinsAA, p.Ser656Ter) and a previously reported nonsense variant (c.2374 C > T, p.Arg792Ter) associated with polydactyly. This study not only elucidates potential mechanisms underlying GLI3-mediated polydactyly syndromes and broadens the variant spectrum of the *GLI3* gene, but also explores the correlation between genotype and phenotype.

### Electronic supplementary material

Below is the link to the electronic supplementary material.

Supplementary Material 1

## Data Availability

No datasets were generated or analysed during the current study.

## Abbreviations

CBP: CREB-Binding Protein domain
CS: Cleavage Site
GCPS: Greig CephaloPolysyndactyly Syndrome
GLI3: GLI family zinc finger 3
GLI1: GLI family zinc finger 1
ZNF141: Zinc finger protein 141
IQCE: IQ domain-Containing Protein E
FAM92A: Family with sequence similarity 92, Member A
DACH1: Dachshund family transcription factor 1
MIPOL1: Mirror-Image polydactyly 1
PITX1: Paired-Like homeodomain 1
IPD: Isolated PolyDactyly
PHS: Pallister-hall syndrome
RD: Repressor domain
SHH: Sonic Hedgehog
SUFU: Suppressor of Fused site
TAD: Transactivation domain
WES: Whole-Exome Sequencing
ZFN: Zinc Finger domain
indel: insertion-deletion
DNA: Deoxyribonucleic Acid
PCR: Polymerase Chain Reaction

## References

[1] Zhang N, Zhong WY, Tian W. Epidemiological characteristics and risk factors of polydactyly. J Clin Orthop Res. 2023;08(01):63–8.

[2] Ahmad Z, Liaqat R, Palander O, Bilal M, Zeb S, Ahmad F, et al. Genetic overview of postaxial polydactyly: updated classification. Clin Genet. 2023;103(1):3–15. 36071556 10.1111/cge.14224

[3] Kelly DM, Mahmoud K, Mauck BM. Polydactyly of the foot: a review. J Am Acad Orthop Surg. 2021;29(9):361–9. 33443388 10.5435/JAAOS-D-20-00983

[4] Chen X, Yuan L, Xu H, Hu P, Yang Y, Guo Y, et al. Novel GLI3 mutations in Chinese patients with non-syndromic post-axial Polydactyly. Curr Mol Med. 2019;19(3):228–35. 30848202 10.2174/1566524019666190308110122

[5] Kalsoom Ue, Klopocki E, Wasif N, Tariq M, Khan S, Hecht J, et al. Whole exome sequencing identified a novel zinc-finger gene ZNF141 associated with autosomal recessive postaxial polydactyly type A. J Med Genet. 2013;50(1):47–53. 23160277 10.1136/jmedgenet-2012-101219

[6] Palencia-Campos A, Martínez-Fernández ML, Altunoglu U, Soto-Bielicka P, Torres A, Marín P, et al. Heterozygous pathogenic variants in GLI1 are a common finding in isolated postaxial polydactyly A/B. Hum Mutat. 2020;41(1):265–76. 31549748 10.1002/humu.23921

[7] Schrauwen I, Giese AP, Aziz A, Lafont DT, Chakchouk I, Santos-Cortez RLP, et al. FAM92A underlies nonsyndromic Postaxial Polydactyly in humans and an abnormal limb and digit skeletal phenotype in mice. J Bone Min Res. 2019;34(2):375–86. 10.1002/jbmr.3594 PMC6489482 30395363

[8] Ullah I, Kakar N, Schrauwen I, Hussain S, Chakchouk I, Liaqat K, et al. Variants in KIAA0825 underlie autosomal recessive postaxial polydactyly. Hum Genet. 2019;138(6):593–600. 30982135 10.1007/s00439-019-02000-0 PMC6724712

[9] Umair M, Shah K, Alhaddad B, Haack TB, Graf E, Strom TM, et al. Exome sequencing revealed a splice site variant in the IQCE gene underlying post-axial polydactyly type A restricted to lower limb. Eur J Hum Genet. 2017;25(8):960–5. 28488682 10.1038/ejhg.2017.83 PMC5567151

[10] Umair M, Palander O, Bilal M, Almuzzaini B, Alam Q, Ahmad F, et al. Biallelic variant in DACH1, encoding Dachshund Homolog 1, defines a novel candidate locus for recessive postaxial polydactyly type A. Genomics. 2021;113(4):2495–502. 34022343 10.1016/j.ygeno.2021.05.015

[11] Kondoh S, Sugawara H, Harada N, Matsumoto N, Ohashi H, Sato M, et al. A novel gene is disrupted at a 14q13 breakpoint of t(2;14) in a patient with mirror-image polydactyly of hands and feet. J Hum Genet. 2002;47(3):136–9. 11954550 10.1007/s100380200015

[12] Klopocki E, Kähler C, Foulds N, Shah H, Joseph B, Vogel H, et al. Deletions in PITX1 cause a spectrum of lower-limb malformations including mirror-image polydactyly. Eur J Hum Genet. 2012;20(6):705–8. 22258522 10.1038/ejhg.2011.264 PMC3355260

[13] Biesecker LG. The Greig Cephalopolysyndactyly syndrome. Orphanet J Rare Dis. 2008;3:10. 18435847 10.1186/1750-1172-3-10 PMC2397380

[14] Tempé D, Casas M, Karaz S, Blanchet-Tournier MF, Concordet JP. Multisite protein kinase A and glycogen synthase kinase 3beta phosphorylation leads to Gli3 ubiquitination by SCFbetaTrCP. Mol Cell Biol. 2006;26(11):4316–26. 16705181 10.1128/MCB.02183-05 PMC1489100

[15] Marigo V, Johnson RL, Vortkamp A, Tabin CJ. Sonic hedgehog differentially regulates expression of GLI and GLI3 during limb development. Dev Biol. 1996;180(1):273–83. 8948590 10.1006/dbio.1996.0300

[16] Riddle RD, Johnson RL, Laufer E, Tabin C. Sonic hedgehog mediates the polarizing activity of the ZPA. Cell. 1993;75(7):1401–16. 8269518 10.1016/0092-8674(93)90626-2

[17] Abdullah YM, Azeem Z, Bilal M, Liaqat K, Hussain S, et al. Variants in GLI3 cause Greig Cephalopolysyndactyly Syndrome. Genet Test Mol Biomarkers. 2019;23(10):744–50. 31573334 10.1089/gtmb.2019.0071

[18] Al-Qattan MM. A novel frameshift mutation of the GLI3 gene in a family with broad thumbs with/without big toes, postaxial polydactyly and variable syndactyly of the hands/feet. Clin Genet. 2012;82(5):502–4. 22428873 10.1111/j.1399-0004.2012.01866.x

[19] Kariminejad A, Ghaderi-Sohi S, Keshavarz E, Hashemi SA, Parsimehr E, Szenker-Ravi E, et al. A GLI3 variant leading to polydactyly in heterozygotes and Pallister-Hall-Like syndrome in a homozygote. Clin Genet. 2020;97(6):915–9. 32112393 10.1111/cge.13730

[20] Radhakrishna U, Bornholdt D, Scott HS, Patel UC, Rossier C, Engel H, et al. The phenotypic spectrum of GLI3 morphopathies includes autosomal dominant preaxial polydactyly type-IV and postaxial polydactyly type-A/B; no phenotype prediction from the position of GLI3 mutations. Am J Hum Genet. 1999;65(3):645–55. 10441570 10.1086/302557 PMC1377970

[21] Dong Y, Du R, Fan LL, Jin JY, Huang H, Chen YQ, et al. Whole-exome sequencing identifies a novel TRPM4 mutation in a Chinese family with atrioventricular block. Biomed Res Int. 2021;2021:9247541. 33959666 10.1155/2021/9247541 PMC8075657

[22] Wang CY, Chen YQ, Jin JY, Du R, Fan LL, Xiang R. A novel nonsense mutation of ABCA8 in a Han-Chinese Family with ASCVD leads to the reduction of HDL-c levels. Front Genet. 2020;11:755. 32760429 10.3389/fgene.2020.00755 PMC7373792

[23] Guo BB, Jin JY, Yuan ZZ, Zeng L, Xiang R. A novel COMP mutated allele identified in a Chinese family with Pseudoachondroplasia. Biomed Res Int. 2021;2021:6678531. 33748277 10.1155/2021/6678531 PMC7960025

[24] Patel R, Singh CB, Bhattacharya V, Singh SK, Ali A. GLI3 mutations in syndromic and non-syndromic polydactyly in two Indian families. Congenit Anom (Kyoto). 2016;56(2):94–7. 26508445 10.1111/cga.12139

[25] Dai P, Akimaru H, Tanaka Y, Maekawa T, Nakafuku M, Ishii S. Sonic hedgehog-induced activation of the Gli1 promoter is mediated by GLI3. J Biol Chem. 1999;274(12):8143–52. 10075717 10.1074/jbc.274.12.8143

[26] Kalff-Suske M, Wild A, Topp J, Wessling M, Jacobsen EM, Bornholdt D, et al. Point mutations throughout the GLI3 gene cause Greig Cephalopolysyndactyly syndrome. Hum Mol Genet. 1999;8(9):1769–77. 10441342 10.1093/hmg/8.9.1769

[27] Tsanev R, Tiigimägi P, Michelson P, Metsis M, Østerlund T, Kogerman P. Identification of the gene transcription repressor domain of Gli3. FEBS Lett. 2009;583(1):224–8. 19084012 10.1016/j.febslet.2008.12.010 PMC2697317

[28] Wang Y, Hao X, Jia X, Ji W, Yuan S, Gnamey EJA, et al. A novel variant of GLI3, p.Asp1514Thrfs*5, is identified in a Chinese family affected by polydactyly. Mol Genet Genomic Med. 2022;10(7):e1968. 35546307 10.1002/mgg3.1968 PMC9266609

[29] Letelier J, Naranjo S, Sospedra-Arrufat I, Martinez-Morales JR, Lopez-Rios J, Shubin N et al. The Shh/Gli3 gene regulatory network precedes the origin of paired fins and reveals the deep homology between distal fins and digits. Proc Natl Acad Sci U S A [Internet]. 2021;118(46). https://pubmed.ncbi.nlm.nih.gov/34750251. 10.1073/pnas.2100575118 PMC8673081 34750251

[30] Veistinen L, Takatalo M, Tanimoto Y, Kesper DA, Vortkamp A, Rice DPC. Loss-of-function of Gli3 in mice causes abnormal frontal bone morphology and premature synostosis of the Interfrontal suture. Front Physiol. 2012;3:121. 22563320 10.3389/fphys.2012.00121 PMC3342524

[31] AlKattan WM, Al-Qattan MM, Bafaqeeh SA. The pathogenesis of the clinical features of oral-facial-digital syndrome type I. Saudi Med J. 2015;36(11):1277–84. 26593159 10.15537/smj.2015.11.12446 PMC4673363

[32] Sczakiel HL, Hülsemann W, Holtgrewe M, Abad-Perez AT, Elsner J, Schwartzmann S, et al. GLI3 variants causing isolated polysyndactyly are not restricted to the protein’s C-terminal third. Clin Genet. 2021;100(6):758–65. 34482537 10.1111/cge.14059

[33] Baas M, Burger EB, van den Ouweland AM, Hovius SE, de Klein A, van Nieuwenhoven CA, et al. Variant type and position predict two distinct limb phenotypes in patients with GLI3-mediated polydactyly syndromes. J Med Genet. 2021;58(6):362–8. 32591344 10.1136/jmedgenet-2020-106948 PMC8142428

[34] Deng H, Tan T, Yuan L. Advances in the molecular genetics of non-syndromic polydactyly. Expert Rev Mol Med. 2015;17:e18. 26515020 10.1017/erm.2015.18

[35] Maffulli N, Margiotti K, Longo UG, Loppini M, Fazio VM, Denaro V. The genetics of sports injuries and athletic performance. Muscles Ligaments Tendons J. 2013;3(3):173–89. 24367777 PMC3838326

[36] Artells R, Pruna R, Dellal A, Maffulli N. Elastin: a possible genetic biomarker for more severe ligament injuries in elite soccer. A pilot study. Muscles Ligaments Tendons J. 2016;6(2):188–92. 27900291 10.32098/mltj.02.2016.04 PMC5115249

[37] Longo UG, Loppini M, Margiotti K, Salvatore G, Berton A, Khan WS, et al. Unravelling the genetic susceptibility to develop ligament and tendon injuries. Curr Stem Cell Res Ther. 2015;10(1):56–63. 25012736 10.2174/1574888X09666140710112535

[38] Kambouris M, Ntalouka F, Ziogas G, Maffulli N. Predictive genomics DNA profiling for athletic performance. Recent Pat DNA Gene Seq. 2012;6(3):229–39. 22827597 10.2174/187221512802717321

[39] Pruna R, Artells R, Lundblad M, Maffulli N. Genetic biomarkers in non-contact muscle injuries in elite soccer players. Knee Surg Sports Traumatol Arthrosc. 2017;25(10):3311–8. 27085366 10.1007/s00167-016-4081-6

[40] Kambouris M, Del Buono A, Maffulli N. Genomics DNA profiling in elite professional soccer players: a pilot study. Transl Med UniSa. 2014;9:18–22. 24809029 PMC4012369

[41] Pruna R, Artells R, Ribas J, Montoro B, Cos F, Muñoz C, et al. Single nucleotide polymorphisms associated with non-contact soft tissue injuries in elite professional soccer players: influence on degree of injury and recovery time. BMC Musculoskelet Disord. 2013;14:221. 23890452 10.1186/1471-2474-14-221 PMC3726514

[42] Clos E, Pruna R, Lundblad M, Artells R, Maffulli N. ACTN3’s R577X single nucleotide polymorphism allele distribution differs significantly in Professional Football players according to their field position. Med Princ Pract. 2021;30(1):92–7. 32492691 10.1159/000509089 PMC7923889

[43] Aicale R, Tarantino D, Maccauro G, Peretti GM, Maffulli N. Genetics in orthopaedic practice. J Biol Regul Homeost Agents [Internet]. 2019;33(2 Suppl. 1). https://pubmed.ncbi.nlm.nih.gov/31169010. 31169010

